# 336. Developing a Protein Signature of Early Latent Tuberculosis Infection.

**DOI:** 10.1093/ofid/ofac492.414

**Published:** 2022-12-15

**Authors:** Charles Bark, Mary N Nsereko, Noah Kiwanuka, Christopher C Whalen, Catherine M Stein, Henry Boom, Harriet Mayanja-Kizza, Sophie Nalukwago, Robert Kakaire, JohnPaul Kasule Jingo, Eustache Paramithiotis

**Affiliations:** MetroHealth Medical Center, Cleveland, Ohio; Uganda CASE Research Clinic, Kampala, Kampala, Uganda; Makerere University, Entebbe, Wakiso, Uganda; University of Georgia, Athens, Georgia; Case Western Reserve University, Cleveland, Ohio; Case Western Reserve University / University Hospitals Cleveland Medical Center, Cleveland, Ohio; MAKERERE UNIVERSITY, KAMPALA, Kampala, Uganda; Uganda CWRU Research Collaboration, Kampala, Luwero, Uganda; University of Georgia, Athens, Georgia; Makerere University, Entebbe, Wakiso, Uganda; CellCarta Biosciences, Montreal, Quebec, Canada

## Abstract

**Background:**

To curb the tuberculosis (TB) epidemic, new cases must be prevented. Preventive therapy is an effective intervention but treating all individuals with latent TB infection (LTBI) based on current diagnostics, tuberculin skin test (TST) and interferon gamma release assay (IGRA), is not feasible. Using proteomic analysis we are developing a peripheral blood marker of LTBI infection that would identify people at high risk for TB progression.

**Methods:**

Plasma was collected in an ongoing TB household contact study in Kampala, Uganda in which TST-/IGRA- are enrolled and followed for IGRA/TST conversion. Longitudinal sample sets (0,6,12 months) from 81 participants including 24 TST/IGRA converters, 29 resisters (remained TST/IGRA negative), and 28 baseline positives (LTBI) underwent proteomic and multiplex cytokine analysis. Proteomic analysis was performed by multiple reaction monitoring mass spectrometry (MRM-MS) using an MRM-MS assay containing 163 host proteins represented by 392 unique peptides, all of which we have described to significantly change during *Mtb* infection in our previous study of household contact converters.

**Results:**

Differential expression of proteins measured by MRM-MS and cytokine assays were assessed. The largest number of significant changes were measured in the LTBI vs Converter comparison. The vast majority of the changes were elevated expression in the converter group compared to the latent group, suggesting that all the biologies affected were induced in the converters (Figure 1). We identified protein panels that were able to predict conversion as in our earlier study. However, the protein panels defined in our earlier study that could distinguish a future converter from a future resister with high fidelity did not have a similar performance in this study.

**Figure 1:**
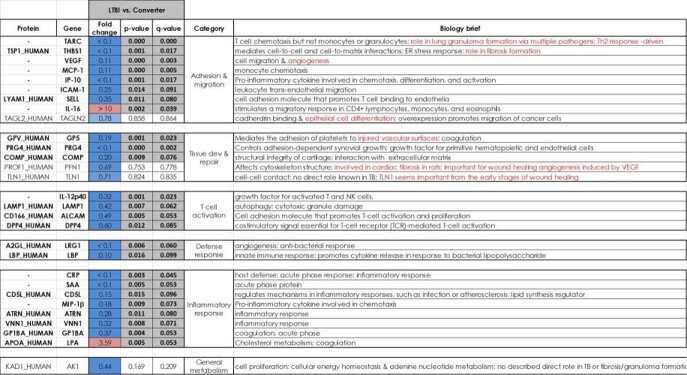
Differential expression analysis of latent TB, LTBI vs. converter. Significant changes by both p-value (set at 0.05) and q-value (a correction for multiple comparisons, set at 0.10).

**Conclusion:**

Peripheral blood protein changes are associated with conversion to LTBI. In this study we found that the protein panels predictive of conversion were different from our previous study. While there are many possible reasons, an important difference between the two studies was the definition of conversion: TST in the older study and QuantiFERON in the current study. We are continuing to investigate this difference and developing time-sensitive protein panels to capture recent conversion.

**Disclosures:**

**Eustache Paramithiotis, PhD**, CellCarta Biosciences: Employee.

